# Birth-related posttraumatic stress disorder and negative childbirth experience related to maternal functioning among adolescent mothers: a cross-sectional study

**DOI:** 10.1186/s12884-023-05717-z

**Published:** 2023-05-22

**Authors:** Fereshteh Vahidi, Mojgan Mirghafourvand, Elaheh Naseri, Solmaz Ghanbari-Homaie

**Affiliations:** 1 Students Research Committee, Tabriz University of Medical Sciences, Tabriz, Iran; 2 Social Determinants of Health Research Center, Tabriz University of Medical Sciences, Tabriz, Iran; 3 Department of Psychology, Faculty of Education and Psychology, University of Tabriz, Tabriz, Iran; 4 Department of Midwifery, Faculty of Nursing and Midwifery, Tabriz University of Medical Sciences, Tabriz, Iran

**Keywords:** PTSD, Teenage, Birth satisfaction, Maternal performance

## Abstract

**Background:**

Adolescent pregnancy is an important issue in terms of reproductive health. Adolescent mothers have to overcome two crises at the same time: motherhood and maturity. Childbirth experience and posttraumatic stress disorder may influence the mother’s perception of her infant and postpartum care behaviors.

**Methods:**

This cross-sectional study was conducted on 202 adolescent mothers referring to health centers in Tabriz and its suburbs between May and December, 2022. Data were collected by PTSD Symptom Scale, Childbirth Experience Questionnaire 2.0, and Barkin Index of Maternal Functioning. The association between childbirth experience, posttraumatic stress disorder and maternal functioning was assessed by multivariate analysis.

**Results:**

After adjusting the effect of socio-demographic and obstetric characteristics, the score of maternal functioning among mothers without posttraumatic stress disorder was statistically significantly higher than mothers with posttraumatic stress disorder diagnosis [β (95% CI) **=** 2.30 (0.39 to 4.20); p = 0.031]. The score of maternal functioning increased with the increase in the childbirth experience score [β (95% CI) **=** 7.34 (3.87 to 10.81); p < 0.001]. The score of maternal functioning among mothers with wanted sex of baby was statistically significantly higher than unwanted sex of baby [β (95% CI) **=** 2.70 (0.37 to 5.02); p = 0.023].

**Conclusion:**

Healthcare professionals should pay special attention to improving maternal functioning among adolescent mothers. One of the important actions can be to create a positive experience of childbirth for avoiding of posttraumatic stress disorder following birth and counseling with mothers who stated sex of fetus is undesired.

## Introduction

The World Health Organization (WHO) defines pregnancy between the ages of 10 and 19 as adolescent pregnancy [1]. One of the most critical health problems in the last century is adolescent pregnancy, which is the leading cause of death for mothers aged 15 to 19 [2, 3]. Globally, the birth rate among adolescents has been reported 42.5 births per 1000 women [4], however, the rate of obstetric complications and mortality is 23% [5]. Adolescent birth in Iran is approximately 27.6 per 1000 women [6].

Childbirth is as a subjective, complex, and multidimensional experience [7]. A meta-analysis found that 4.7% of women were affected by birth-related post-traumatic stress disorder (PTSD) [8]. PTSD can occur due to difficult birth and possible injuries among adolescent mothers [8, 9]. One third of American adolescent mothers have evaluated their birth as traumatic, and half of them have reported symptoms of trauma immediately after giving birth [9]. Most mental disorders start during adolescence and they may continue until adulthood [10]. Probably, adolescent pregnant women are afraid of health care provider’s behavior, discrimination, experiencing disrespect and exclusion, insufficient provision of information about pregnancy and labour, and interventions during labour and childbirth [11]. The fear is probably due to the stage of maturity or insufficient training of adolescents so that most adolescent women request for more pain relief and emotional support during labour [12]. On the other hand, underdeveloped pelvis in adolescents makes them at risk for Cephalo Pelvic Disproportion (CPD) and finally cesarean delivery [13]. Dystocia and cesarean birth itself are more likely to result in PTSD [14].

Adolescent pregnancy is an important issue in terms of reproductive health [15] which is associated with accepting new roles and responsibilities and physical, social, and psychological changes [16]. Adolescent mothers have to overcome two crises at the same time: motherhood and maturity [17]. These mothers may have poor quality maternal functioning due to insufficient knowledge of how to care for the child [18].

Childbirth experience can influence the mother’s perception of her infant and postpartum care behaviors [19]. Maternal negative perception is associated with her poor behavioral skills and sense of competence [20]. According to our knowledge, the relationship between post-traumatic stress disorder, childbirth experience and maternal functioning has been investigated in limited studies and in adult mothers. Therefore, this study investigated the relationship between childbirth experience and PTSD with maternal functioning in Iranian adolescent mothers.

## Materials and methods

### Study design and participants

This cross-sectional study was conducted on 202 adolescent mothers referring to health centers in Tabriz and its suburbs. The inclusion criteria were: living in Tabriz city and suburbs; age between 10 and 19; and at least one month and a maximum of 3 months have passed since giving birth. The non-inclusion criteria were: having a mental disability; being deaf and dumb; having multiple pregnancies; a known abnormality in the baby; a history of depression based on the mother’s self-report and medical records; taking antidepressants; the occurrence of an important stressful event such as the death of a first-degree relative within three months before data collection.

### Sampling

Due to the low prevalence of adolescent pregnancy, census sampling was done from all urban and suburb health centers in Tabriz city, Iran. The list of adolescent mothers (1 to 3 months after giving birth) along with their phone numbers were received from respective health center by researcher (MSc midwifery student- first author). Mothers were checked in terms of the eligibility criteria according telephone interview and review of electronic health records by researcher. After ensuring the eligibility criteria and willingness to cooperate, the mother was asked to come to the health center on the day of care receipt postpartum to complete the informed consent form and questionnaires. Due to the covid-19 pandemic and the reluctance of some mothers to visit in person, mothers who had access to the internet and social networks were requested to complete the relevant forms online.

### Setting

Tabriz city has 92 urban and rural health centers. Some health centers had no adolescent mothers, consequently the number of urban centers that made up the target group of our study was 38 centers and the number of suburban centers was 26 centers.

### Data collection scales

Data collection tools in this study included socio- demographic and obstetric checklists, Childbirth Experience Questionnaire 2.0 (CEQ 2.0), PTSD Symptom Scale (PSS-I), and Barkin Index of Maternal Functioning (BIMF).

Socio- demographic and obstetric checklists.

This checklist contains questions such as maternal age, husband age, occupation, husband occupation, maternal educational level, husband educational level, forced marriage, life satisfaction, income adequacy, type of residence place, gestational age at birth time, parity, dissatisfaction with pervious birth, unintended pregnancy, unwanted sex of baby from viewpoint of mother, physical violence during pregnancy, having emotional support from family, complications during pregnancy (preeclampsia, diabetes, hypothyroidism, anemia), doula support, birth attendant (obstetrician professor, obstetrician resident, midwifery/ midwifery student), type of hospital for birth (teaching, organizational, private).

Childbirth Experience Questionnaire 2.0 (CEQ 2.0).

This questionnaire contains 23 questions with following sub-scales: “own capacity”, “professional support”, “safety”, and “participation”. The total score range of the CEQ2.0 is between 1 and 4, and low average scores indicate a more negative childbirth experience. CEQ2.0 has been psychometrically evaluated in Iran by Ghanbari et al. Cronbach’s alpha coefficient was 0.93, and the intraclass correlation coefficient (ICC) was 0.97 for the entire questionnaire and 0.81 and above for the sub-scales, which indicates an acceptable agreement [21].

PTSD Symptom Scale (PSS-I).

This questionnaire consists of 17 questions that grade the severity of symptoms based on the Diagnostic and Statistical Manual of Mental Disorders- Forth version (DSM-IV) for diagnosing post-traumatic stress using a Likert scale. The presence of one or more of the symptoms of “re-experiencing”, three or more of the symptoms related to “avoidance”, and two or more of the factors related to “Increased arousal”, post-traumatic stress disorder was diagnosed. The PSS-I also shows the severity of post-traumatic stress. PTSD severity for each symptom domain can be calculated by summing the scores in each domain. The range of scores is from 0 to 51. Cronbach’s alpha for the Persian version is 0.88, and the test-retest reliability has been reported to be equal to 1, indicating the score’s stability and internal consistency using PSS-I [22].

Barkin Index of Maternal Functioning (BIMF).

The BIMF contains 17 questions with following sub-scales: “Mom’s need” and “Mom’s competency”. The responses are completely disagree (score 0), disagree (score 1), somewhat disagree (score 2), did not make a decision (score 3), somewhat agree (score 4), agree (score 5) and completely agree (score 6). The overall score ranges from 0 to 102, with higher scores indicating better functioning. The Persian version of the BIMF is a valid and reliable tool (Cronbach’s alpha coefficient = 0.88; ICC = 0.85) [23, 24].

### Sample size

The sample size was calculated based on the Havizari et al.’s study and taking into account the correlation coefficient of childbirth experience and maternal functioning equal to -0.32 and two sided α = 0.05 and power = 99% equal to 170. The final sample size was calculated as 202 women with considering a 20% attrition [25]. A total of 202 were included in the study between May and December, 2022.

### Ethics approval and consent to participate

All methods were carried out in accordance with Helsinki declaration. The study has been approved by the Ethics Committee of Tabriz University of Medical Sciences, Tabriz, Iran (Code: Code: IR.TBZMED.REC.1400.1116). Written informed consent was obtained from all participants. Any participant who had aged below 16 or illiterate, informed consent was taken from their legal guardian.

### Data analysis

Data were analyzed using Version 24.0 software for Windows (IBM Inc., Armonk, NY, USA). For quantitative data with normal distribution, mean (standard deviation) and data without normal distribution, median (25th, 75th percentile) was reported. In univariate analysis, to determine the relationship between socio-demographic and obstetric variables with maternal functioning, Pearson correlation, Spearman’s correlation, one-way ANOVA, and independent-samples t tests were used. In the next step, the variables with P-value < 0.1 were entered into the multivariate analysis, and by adjusting the effect of these variables, the association between childbirth experience, posttraumatic stress disorder and maternal functioning was explored.

## Results

A total of 202 mothers out 258 eligible adolescent mothers (response rate = 78%) from 64 urban and suburban areas of Tabriz, East Azerbaijan province, Iran, were included in the study between May and December, 2022. The majority of mothers (97%) completed the scales in person. The mean (Standard Deviation) age and marriage age of the participants were 17.8 (1.2) and 14.9 (1.7) years, respectively. Approximately 14.4% of the mothers stated that they had a forced marriage. There was no statistically significant relationship between socio-demographic characteristics and maternal functioning. Only, there was statistically significant relationship between residence places in term of maternal functioning (p = 0.023) (Table 1).

**Table 1:** Relationship between socio-demographic characteristics with maternal functioning among adolescent mothers (n = 202)

Characteristics	Number (Percent)	Relationship with maternal functioning	
		Mean (SD)	p
**Maternal age (years), Mean (SD)**	17.8 (1.2)	-0.07^a^	0.232^b^
**Husband age (years), Mean (SD)**	26.5 (3.4)	0.03^a^	0.704^b^
**Marriage age (years), Mean (SD)**	14.9 (1.7)	-0.14^a^	0.052^b^
**Maternal Educational level**			0.609^c^
Illiterate/ elementary	27 (13.4)	85.8 (5.6)	
Secondary/ high school	152 (75.2)	86.6 (5.2)	
Diploma	23 (11.4)	85.6 (6.1)	
**Husband Educational level**			0.940^c^
Illiterate or elementary	18 (8.9)	85.9 (6.3)	
Secondary or high school	78 (38.6)	86.2 (5.2)	
Diploma	87 (43.1)	86.6 (5.4)	
Academic	19 (9.4)	86.7 (5.4)	
**Maternal Occupation**			0.302^d^
Housewife	200 (99)	86.4 (5.4)	
Self-employed	2 (1)	82.5 (6.3)	
**Husband Occupation**			0.682^d^
Worker	43 (21.3)	86.1 (5.8)	
Self-employed	159 (78.7)	86.5 (5.2)	
**Forced marriage**			0.679^d^
Yes	29 (14.4)	86.3 (5.5)	
No	173 (85.6)	86.8 (4.5)	
**Life satisfaction**			0.32^d^
Relatively	30 (14.9)	85.5 (5.5)	
Completely	172 (85.1)	86.5 (5.3)	
**Income adequacy**			0.289^c^
Inadequate	6 (3.0)	83.6 (6.9)	
Relatively adequate	114 (56.4)	86.2 (5.2)	
Completely adequate	82 (40.6)	86.9 (5.5)	
**Residence place**			**0.023** ^c^
Personal	97 (48.0)	87.4 (5.0)	
Rental	56 (27.7)	86.4 (5.2)	
Relative	49 (24.3)	87.5 (0.7)	

The data indicate frequency (percent). P < 0.05 indicates significance difference

^a^ r; ^b^ Pearson correlation test; ^c^ One-Way ANOVA; ^d^ Independent-Samples T Test

The majority of mothers (93%) were primiparous. Approximately 17.6% of mothers were dissatisfied with their previous childbirth. Nearly a quarter of mothers (20.8%) stated that their pregnancy was unwanted. Only about three-quarters of mothers (75.2%) had emotional support during pregnancy. More than a third of mothers (39.5%) had experienced one of the complications in the postpartum period (bleeding, infection, and thrombosis). Approximately 12.4% of mothers had an emergency cesarean section. There was no statistically significant relationship between obstetric characteristics and maternal functioning. Only, there was statistically significant relationship between physical violence during pregnancy in term of maternal functioning (p = 0.004) (Table 2).

**Table 2:** Relationship between obstetric characteristics with maternal functioning among adolescent mothers (n = 202)

Characteristics	Number (Percent)	Relationship with maternal functioning	
		Mean (SD)	p
**Gestational age at birth time (Weeks), Mean (SD)**	38.3 (1.7)	-0.05	0.491^a^
**BMI (kg/m2), Mean (SD)**	23.2 (4.5)	-0.06	0.381^a^
**Parity**			0.209^b^
1	187 (92.6)	86.3 (5.5)	
2	15 (7.4)	88.1 (3.5)	
**History of abortion**			0.166 ^b^
Yes	24 (11.9)	85.0 (4.7)	
No	178 (88.1)	86.6 (5.4)	
**Dissatisfaction with pervious birth among multiparous women**			0.342^b^
Yes	3 (20.0)	83.6 (8.1)	
No	12 (80.0)	86.7 (4.3)	
**Unintended pregnancy**			0.314^b^
Yes	42 (20.8)	85.7 (5.6)	
No	160 (79.2)	86.6 (5.3)	
**Unwanted sex of baby**			0.066^b^
Yes	19 (9.4)	84.2 (5.9)	
No	183 (90.6)	86.6 (5.3)	
**Physical violence during pregnancy**			**0.004** ^**b**^
Yes	7 (3.5)	80.7 (6.6)	
No	195 (96.5)	86.6 (5.2)	
**Having emotional support from family**			0.118^b^
Yes	152 (75.2)	86.7 (5.3)	
No	50 (24.8)	85.4 (5.6)	
**Complications during pregnancy** ^**c**^			0.976^b^
Yes	32 (39.5)	85.0 (4.5)	
No	49 (60.5)	85.1 (6.1)	
**Doula support**			0.766^b^
Yes	8 (4.0)	87.0 (5.8)	
No	194 (96.0)	86.4 (5.4)	
**Birth attendant**			0.803^d^
Obstetrician	61 (30.2)	86.8 (4.9)	
Obstetrician resident	12 (5.9)	85.4 (4.2)	
Midwifery/ midwifery student	129 (63.9)	86.3 (5.7)	
**Type of birth**			0.495^d^
Vaginal	56 (27.7)	86.9 (5.0)	
Vaginal + episiotomy	121 (59.9)	86.0 (5.6)	
Emergency cesarean section	25 (12.4)	87.1 (5.2)	
**Type of hospital for birth**			0.319^d^
Teaching	165 (81.7)	86.3 (5.2)	
Organizational	10 (5.0)	84.7 (5.8)	
Private	27 (13.4)	87.5 (6.2)	
**Skin to skin contact**			0.127^b^
Yes	168 (83.2)	86.7 (5.4)	
No	34 (16.8)	85.1 (5.1)	
**Postpartum complication** ^**e**^			0.051^b^
Yes	26 (13.0)	84.0 (5.6)	
No	176 (871)	86.8 (5.2)	
**ICU admission** ^**f**^			0.154^b^
Yes	6 (3.0)	83.3 (7.3)	
No	196 (97.0)	86.5 (5.3)	
**NICU admission** ^**g**^			0.404^b^
Yes	44 (21.8)	87.0 (4.9)	
No	158 (78.2)	86.2 (5.5)	
**Baby weight (g)**	3137.7 (473.8)	0.016	0.824^b^
**Baby sex (male)**	110 (54.5)	87.0 (5.5)	0.092^b^
Female	92 (45.5)	85.7 (5.1)	
**Infant disease during postpartum** ^**h**^			0.885^b^
Yes	128 (63.4)	86.3 (5.7)	
No	74 (36.6)	86.5 (4.8)	

^a^ Pearson correlation test; ^b^ Independent-Samples T Test; ^c^ Preeclampsia, Diabetes, Hypothyroidism, Anemia; ^d^ One-Way ANOVA; ^e^ Hemorrhage, Infection, Thrombosis; ^f^ Intensive Care Unit; ^g^ Neonatal Intensive Care Unit; ^h^ Prematurity (9.4%), Icteric (19.8%), Respiratory (17.3%), Colic/ Reflux (16.9%)

The mean (SD) of the overall maternal functioning score was 86.4 (5.4) from a possible score of 0 to 120. The mean (SD) of mom’s need (score 0 to 18) and mom’s competence (score 0 to 84) subscales were 14.1 (2.7) and 72.3 (3.8), respectively. The mean (SD) of the overall childbirth experience score was 2.7 (0.2) from a possible score of 1 to 4. The mean (SD) of the total score of birth-related posttraumatic stress disorder from a possible score of 0 to 151 was 4.6 (6.2). The median (percentile 25, 75) of the total score of PTSD was 2 (0, 6.2). The number (percent) of women with PTSD criteria was 37 (18.3%) (Table 3).

**Table 3:** Relationship of maternal functioning with birth experience and post-traumatic stress disorder among adolescent mothers (n = 202)

Variables	Mean (SD)	Maternal Functioning	Mom’s Need	Mom’s Competency
	r (p-value) ^a^			
**Total score of PTSD (0–51)** ^**b**^	2 (0, 6.2) ^c^	-0.28 (< 0.001)	-0.14 (0.037)	-0.30 (< 0.001)
Re-experiencing (0–12)	0 (0, 2.0) ^c^	-0.28 (< 0.001)	-0.16 (0.017)	-0.28 (< 0.001)
Avoidance (0–21)	1 (0, 3.0) ^c^	-0.23 (0.001)	-0.13 (0.050)	-0.24 (< 0.001)
Increased arousal (0–18)	1 (0, 3.0) ^c^	-0.23 (0.001)	-0.09 (0.186)	-0.25 (< 0.001)
**PTSD, N (%)**	37 (18.3%)			
**Total score of CEQ (1 to 4)** ^**d**^	2.7 (0.2)	0.33 (< 0.001)	0.18 (0.007)	0.33 (< 0.001)
Own capacity (1 to 4)	2.6 (0.2)	0.32 (< 0.001)	0.20 (0.003)	0.31 (< 0.001)
Participation (1 to 4)	2.9 (0.2)	0.07 (0.303)	0.04 (0.516)	0.07 (0.325)
Perceived safety (1 to 4)	2.5 (0.3)	0.28 (< 0.001)	0.12 (0.067)	0.30 (< 0.001)
Professional support (1 to 4)	3.7 (0.3)	0.18 (0.008)	0.18 (0.008)	0.10 (0.146)
**BIMF (0-102)** ^**e**^	86.4 (5.4)	-		
Mom’s need (0–18)	14.1 (2.7)	-		
Mom’s competency (0–84)	72.3 (3.8)	-		

^a^ Analysis was performed based on Pearson except PTSD in which was based on Spearman’s correlation; ^b^ Post-Traumatic Stress Disorder; ^c^ Median (p 25, p75); ^d^ Childbirth Experience Questionnaire; ^d^ Barkin Index of Maternal Functioning

According to the Spearman test, there was an inverse, moderate and significant correlation between the overall score of PTSD and maternal functioning (r= -0.28; p < 0.001). Also, there was a direct, moderate, and significant correlation between the overall score of childbirth experience and maternal functioning (r = 0.33; p < 0.001). All childbirth experience subdomains except participation had a significant correlation with maternal functioning (p < 0.001) (Table 3).

After adjusting the effect of socio-demographic and obstetric characteristics, the score of maternal functioning among mothers without PTSD was statistically significantly higher than mothers with PTSD diagnosis [β (95% CI) **=** 2.30 (0.39 to 4.20); p = 0.031]. The score of maternal functioning increased with the increase in the childbirth experience score [β (95% CI) **=** 7.34 (3.87 to 10.81); p < 0.001]. The score of maternal functioning among mothers with wanted sex of baby was statistically significantly higher than unwanted sex of baby [β (95% CI) **=** 2.70 (0.37 to 5.02); p = 0.023] (Table 4).

**Table 4:** The relationship of maternal functioning with birth experience and post-traumatic stress disorder among adolescent mothers (n = 202)

Characteristic	β (95% CI)	p ^a^
**Birth-related Posttraumatic Stress Disorder (ref: Yes)**		
No	2.30 (0.39 to 4.20)	**0.031**
**Childbirth Experience**	7.34 (3.87 to 10.81)	**< 0.001**
**Marriage age**	-0.27 (-0.68 to 0.13)	0.188
**Violence (ref: No)**		
Yes	-3.07 (-6.99 to 0.83)	0.123
**Sex of baby (ref: Female)**		
Male	0.53 (-0.82 to 1.89)	0.440
**Postpartum complication (ref: Yes)**		
No	1.44 (-0.62 to 3.52)	0.170
**Unwanted baby sex (ref: Yes)**		
No	2.70 (0.37 to 5.02)	**0.023**
**House (ref: Relative)**		
Personal	1.19 (-0.53 to 2.93)	0.175
Rental	1.08 (-0.81 to 2.98)	0.262

^a^ Analysis based on General Linear Model. Adjusted R Squared = 0.275

## Discussion

This study was conducted to assess the related factors of maternal functioning among adolescent mothers. According to the results obtained from the study, the mean (SD) of the overall maternal functioning score was 86.4 from a possible score of 0 to 120. The mean (SD) of the overall childbirth experience and birth-related posttraumatic stress disorder score were 2.7 and 4.6, respectively. There was a statistically significant relationship between PTSD, childbirth experience and unwanted baby sex with maternal functioning.

The mean of the overall maternal functioning score was 86.4. According the result of a cross-sectional study among Iranian adult mothers, the overall maternal functioning score was 97.4. The overall maternal functioning score among adult mothers was higher than adolescent mothers [25]. According to the studies that examined the mother’s functioning during the transition to this role, adolescent mothers were challenged in taking care of the infant due to a lack of knowledge about the infant [26]. Adolescents have had less responsibility throughout their lives, so it can be difficult for them to fulfill the responsibilities of parenting [18]. Another study between six Black racial identity clusters conducted to investigate maternal functioning among black women (18 to 41 years), there was a significant relationship between a mother’s attachment and education level with a mother’s function [27]. In a study in Italy has been showed that adolescent mothers have experienced more maltreatment from their parents during their childhood than adult mothers, which will affect their parenting behavior as well [28].

In present study, the mean of the overall childbirth experience score was 2.7. According to the results of a study conducted in London, adolescent mothers feel helpless and vulnerable after giving birth and needed help to meet their needs [29]. In another study in Iran among adult mothers, the mean of the overall childbirth experience score was 2.7 which is consistent with the results of this study [30].

The mean of the total score of birth-related posttraumatic stress disorder was 4.6 and the prevalence of PTSD was 18.3%. In a study conducted in Brazil, the experience of childbirth was compared between adults and adolescents. Adolescents were less satisfied with childbirth and the childbirth experience was not as they expected, while according to the majority of adult mothers, the childbirth experience was as they expected [31]. The prevalence of birth-related posttraumatic stress disorder among Iranian adult mothers was 16.3% in which is lower than the adolescent mothers [32]. A longitudinal study by Anderson et al. (2010) among 85 adolescent mothers aged 13 to 19 in America reported that one third of adolescents perceived their birth as traumatic and half of them showed signs of the impact of trauma. Factors affecting the experience included marital status, fear of losing control, fear of death, and sexual partner violence. Also, the assessment of childbirth and symptoms were among the factors affecting the impact of trauma [9]. In Anderson et al.‘s study, in contrast to our study, the rate of PTSD among adolescents’ mothers were high (50%) due to association with traumatic childhood events such as violence and sexual assault. The low percentage of PTSD in this study could be because traumatic event and maternal depression were the exclusion criteria. On the other hand, the role of being mother in teenagers can be challenging due to puberty and the physical and emotional changes that result [33].

In this study, there was a statistically significant relationship between childbirth experience and PTSD with maternal functioning. Although the relationship between postpartum psychological disorders and several maternal and child outcomes has been investigated, the evidence on the relationship between these disorders and maternal functioning is very limited. In consistent with our results, in the study conducted by Havizari et al. among adult mothers, there was a significant correlation between mental health and childbirth experience with maternal functioning. A study by Holopainen et al. in the Netherlands among women aged 15 to 43 showed that childbirth experience at 3 months postpartum was significantly related to maternal stress. However, the childbirth experience was not directly or indirectly through mother’s stress related to the mother-infant attachment [34]. The probable reason for the inconsistency between this study and Holopainen et al. study could be that maternal functioning is beyond maternal attachment. Also, differences in methodology and setting can be other possible reasons. The results of a study by Adam showed that maternal stress is not a strong predictor of mother behavior or mother and child interaction [35]. However, in Adams’ study, the Parent-Child Early Relational Assessment questionnaire has been used. It is mostly designed to assess parental sensitivity, which is different from the Barkin Questionnaire.

In a systematic review, the relationship between PTSD and child outcomes has been investigated. Based on the results of this study, there is an inverse relationship between mother’s PTSD and mother- infant attachment and child’s behavior. Evidence regarding the association of maternal PTSD with breastfeeding, infant weight gain, sleep, and social-emotional development has been insufficient [36].

Postpartum stress and trauma can affect not only the mother’s mental health, but also the mother’s behavior, her relationship with the infant, and even neglect of the child [37, 38]. One of the possible reasons for the findings of the present study may be that poor mental health can lead to less sensitivity and responsiveness of the mother to the child’s needs [39, 40].

The score of maternal functioning among mothers with wanted sex of baby was statistically significantly higher than unwanted sex of baby. Probably, the unwanted sex of the fetus, especially if father do not want it, can lead to unfavorable performance of the mother due to cultural reasons and insufficient received support from the husband [41]. In a study in Indonesia has been showed that the prevalence of postpartum depression is approximately 60% among adolescent mothers and the sex of baby was a potential risk factor for postpartum depression. Postpartum depression has significant impact on the mother’s confidence to care for her baby [42]. Therefore, it’s possible that mothers with unwanted sex of baby had poor maternal functioning.

### Strength and limitations

Sampling was done from urban and rural health centers, and during the interview, it was found that mothers received delivery services from public, private, and teaching hospitals, which can be generalized to similar populations in different cities. Also, there were types of delivery such as vaginal delivery, delivery with episiotomy, and emergency cesarean section in this study. The data collection method in our research is a census, which is one of the positive points of the research. One of the limitations of this study was the relatively low response rate.

It is suggested that this study be conducted longitudinally and prospectively, and after the initial screening, the final diagnosis will be made by a psychiatrist and the treatment of these mothers will be considered. Based on this study, it is recommended that psychologists provide adequate training about this mental disorder to vulnerable adolescents during pregnancy, because PTSD can negatively affect the physical health of the mother and child, as well as maternal functioning. Health providers should also play a role in screening of the PTSD and traumatic childbirth experience to prevent poor maternal functioning.

## Conclusion and implications for practice

There is a significant reverse relationship between post-traumatic stress disorder related to birth and the maternal functioning. Also, by examining the childbirth experience and the mother’s functioning, a direct relationship was found between them. Professional support during labour and childbirth, considering the vulnerability and the need for more support at this age, can create positive memories in the minds of these mothers and reduce the occurrence of postpartum stress disorder. Also, with social support for teenagers and pregnancy prevention at this age, physical and mental harm to the mother and baby can be prevented.

## Data Availability

The datasets used and analysed during the current study available from the corresponding author on reasonable request.

## Abbreviations

CPD: Cephalo Pelvic Disproportion
CEQ 2.0: Childbirth Experience Questionnaire 2.0
PSS-I: PTSD Symptom Scale I
BIMF: Barkin Index of Maternal Functioning
